# The investigation of the NH_3_-SCR performance of a copper-based AEI-CHA intergrown zeolite catalyst

**DOI:** 10.3389/fchem.2022.1069824

**Published:** 2022-11-30

**Authors:** Hongling Ye, Kai Ren, Pengfei Wang, Lin Wang

**Affiliations:** ^1^ School of Mechanical and Vehicle Engineering, Bengbu University, Bengbu, Anhui, China; ^2^ School of Mechanical and Electronic Engineering, Nanjing Forestry University, Nanjing, China

**Keywords:** intergrown structure, copper-based zeolite catalyst, selective catalytic reduction, nitrogen oxides, low-temperature hydrothermal deactivation

## Abstract

This work prepared an ISAPO-34/SAPO-18 intergrown zeolite using phosphate organoamine as the structure guiding agent. Physical-chemical characterizations by XRD, SEM, TG, and BET showed that the SAPO-34/SAPO-18 presents a cross-stacked cubic block-like microscopic morphology, with characteristic diffusive diffraction peaks at 2θ = 16–18° and 30–33° and a specific surface area of 557 m^2^ g^−1^. The series of copper-based catalysts prepared from SAPO-34/SAPO-18 showed a shift of the active temperature window to a lower temperature with increasing copper content. Moreover, the Brønsted acid site decreased significantly due to copper ion exchange and zeolite structure framework damage. Among them, the 1.2 wt% sample showed the widest active temperature window, with a T_90_ range of 175–435°C. After low-temperature hydrothermal aging treatment, the zeolite structure was eroded and the catalyst activity deteriorated significantly.

## Introduction

Due to the recent increasing seriousness of environmental pollution, theoretical studies have explored methods to relieve this phenomenon based on two-dimensional catalysts (Ren et al., 2022a; Ren et al., 2022b; Ren et al., 2022c; Ren et al., 2022d; Zhang et al., 2022). The gas emissions of internal combustion engines have produced considerable levels of pollution. China steadily ranks first worldwide in the production and sales of internal combustion engines, with domestic sales reaching 46.813 million in 2020. Among these, diesel internal combustion engines comprised 6.341 m units, accounting for 13.5% of the total internal combustion engine sales and showing a steady growth trend. Given this trend, the challenge of exhaust pollutant emission is increasingly prominent and seriously threatens the sustainable development of atmospheric ecological environments. The diesel vehicle emission standards in China mainly follow European emission regulations, for which selective catalytic reduction (SCR) technology provides a necessary way for diesel vehicles to meet VI emission standards (Han et al., 2019). The core catalyst required by ammonia selective catalytic reduction (NH_3_-SCR) technology, an internationally recognized efficient technology for nitrogen oxides (NO_X_), is shifted from a vanadium, tungsten, and titanium system (Yang et al., 2014) to a zeolite catalyst system (Chen et al., 2022). The latter is usually composed of zeolite as a carrier of active metal elements (Zheng et al., 2020; Jin et al., 2022; Yuan et al., 2022). The common zeolite skeleton configurations include MFI (Yuan et al., 2016), AEI (Wu et al., 2022), BEA (Lin et al., 2018), LTA (Lin et al., 2021), CHA (Wang et al., 2015; Bergmana et al., 2020; Sun et al., 2020; Zhang et al., 2021a; Bello et al., 2022), AFX (Li et al., 2022a), etc. The zeolite SCR catalyst (Tsukamoto et al., 2019) represented by CHA has advantages including good low-temperature activity, a wide active temperature window, high nitrogen selectivity, green environmental protection due to its unique micropore structure, and suitable surface acidity (Xu et al., 2020; Wang et al., 2021; Zhang et al., 2022a). In recent years, in-depth studies have evaluated the formula, performance, and mechanism of an SCR catalyst with single-structure zeolite. The corresponding intellectual property rights are owned by foreign companies. Polycrystalline/mixed crystal/intergrown zeolite SCR catalysts also show excellent catalytic activity and durability. Zhang et al. (2021b) reported greater catalytic activity, hydrothermal stability, and sulfur aging resistance for a Cu/SAPO-18/34 intergrown zeolite catalyst compared to those for Cu/SAPO-18 and Cu/SAPO-34. As a typical representative, SAPO-18/34 zeolite is a new type of zeolite (Boruntea et al., 2019; Tsuchiya et al., 2020; Li et al., 2022b) composed of AEI and CHA skeleton structure units in stacking faults. It has both the pore canals and acidity of the two crystal phase structures, which usually show better catalytic performance than a single zeolite. Zhao et al. (2016) used triethylamine and N, N-diisopropyl ethylamine as double templates to prepare AEI/CHA intergrown SAPO zeolite and its catalyst, which not only increased the catalytic activity but also significantly reduced the carbon deposition rate. This dual template method is the most common way to prepare symbiotic molecular sieves. However, compared to single templates, it is more difficult to prepare molecular sieves with double templates, and the effects of the proportion, distribution, and chemical state of the templates on the synthesis are more complex. Therefore, the efficient preparation of AEI/CHA symbiotic molecular sieves requires a template.

The present study used phosphate organic amine (Li et al., 2022c) as a template and phosphorus source in a synthesis environment at a pH of 6–7. A SAPO-34/SAPO-18 intergrown zeolite with a hydrogen API-CHA structure was directly prepared and its physicochemical properties and catalytic activity were analyzed by characterization methods including XRD, SEM, and BET. The effects of factors such as active component content and low-temperature hydrothermal inactivation on the performance were studied to provide a reference for the performance research and application of intergrown zeolite SCR catalysts.

## Experimental methods

### Reagents

The reagents included SAPO-18/SAPO-34 zeolite (self-made, SiO_2_:P_2_O_5_:Al_2_O_3_ = 1:3.74:3.81); copper nitrate (analytically pure, Shanghai McLean Biochemical Technology Co., Ltd.); nitric acid (analytically pure, Tianjin Kemio Chemical Reagent Co., Ltd.); deionized water (self-made).

### Preparation of the zeolite catalyst

A 0.1N copper nitrate solution was prepared, 200 g of which was weighed and placed in a 500 ml beaker. Next, 10 g of SAPO-18/SAPO-34 zeolite was added to the solution. Nitric acid was then added until pH = 3, stirred, and reacted in a water bath at 80°C. A series of copper-based catalysts were prepared. Those with copper contents of 0.3wt%, 0.8wt%, and 1.2wt% were labeled as 0.3, 0.8, and 1.2, respectively.

### Characterization of the zeolite catalysts

XRD characterization was performed using a SmartLab SE X-ray diffractometer (Rigaku Corporation) to analyze the crystal structures of the samples. SEM characterization was performed using an Apero-Lowvac high-resolution field emission scanning electron microscope (Thermo Fisher) to observe the sample microstructure. BET characterization was performed using an ASAP 2460 specific surface area and porosity analyzer (Micrometrics) to analyze the specific surface area, pore volume, and pore size of the test samples. NH_3_-TPD characterization was performed using an AutoChem II 2920 chemical adsorption instrument (Micrometrics) to analyze the surface acidity characteristics of the samples. TG-DSC characterization was performed using a STA449F5 Jupiter-type synchronous thermal analyzer (NETZSCH) to assess the mass and heat changes of the samples at increasing temperatures.

### Evaluation of the catalyst activities

**FIGURE 1:**
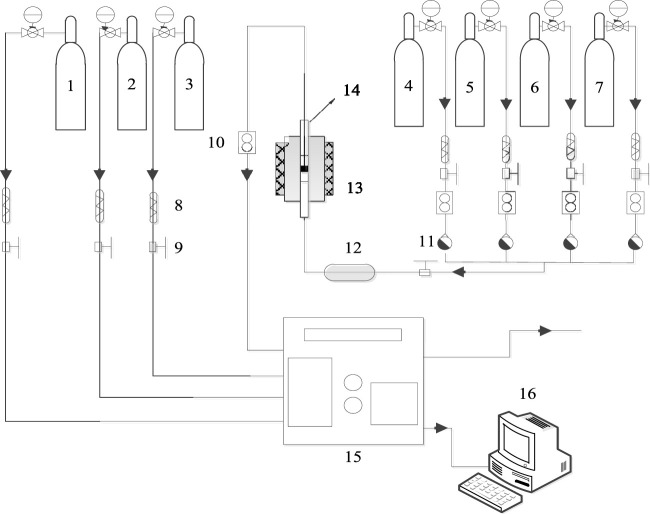
Equipment diagram for the evaluation of the activity of the NH_3_-SCR catalyst. 1. Nitric oxide. 2. Ammonia. 3. Nitrogen. 4. Oxygen. 5. Propane. 7. Other Gases. 8. Filter. 9. Globe valve. 10. Mass flowmeter. 11. One-way valve. 12. Mixer. 13. Heating furnace. 14. Reactor. 15. Gas analyzer. 16. Computer.

The catalytic performance was tested in a miniature fixed-bed activity evaluation device, as shown in Figure 1. For these tests, 5 g catalyst powder was fully ground to prepare a 40–60 mesh sample and placed in a quartz reaction tube with a 15 mm inner diameter. Both ends were sealed with quartz cotton to form a catalyst bed. The evaluation device comprised a simulation gas distribution system, a programmed heating device, and a gas analyzer. The simulated tail gas composition was as follows: [NO] = 500 PPM, [NH_3_] = 500 PPM, [H_2_O] = 10 vol.%, [O_2_] = 10 vol.%, with N_2_ as the equilibrium gas, and an airspeed of 30,000 h^−1^ (default conditions). The NO conversion rate, NH_3_ conversion rate, N_2_O content, and N_2_ selectivity were calculated according to the following formula:

$$x_{NO}=\frac{n_{NO_{in}}-n_{NO_{out}}}{n_{NO_{in}}}\times 100\%$$ (1)

$$s_{N_{2}}=\frac{n_{NH_{3in}}-n_{NH_{3out}}+n_{NO_{in}}-n_{NO_{out}}-n_{NO_{2out}}-2n_{N_{2}O_{out}}}{n_{NH_{3in}}+n_{NO_{in}}-n_{NO_{out}}}\times 100\%$$ (2)

where NO _(out)_, NO_2 (out)_, N_2_O _(out)_, and NH_3 (out)_ are the outlet concentrations of NO, NO_2_, N_2_O, and NH_3_ and NO _(in)_ and NH_3 (in)_ are the inlet concentrations of NO and NH_3_.

The formula for the heating program *T* was as follows:

$$T=\frac{1}{12}\times t+25$$ (3)

where the unit of the *T* is °C; *t* represents the time with the unit of second.

### Low-temperature hydrothermal aging treatment

To investigate the hydrothermal stability of the SAPO-18/SAPO-34 zeolite at low temperature, an SCR catalyst with 1.2% copper content was aged for 100 h at 90°C under 10% water vapor at a space speed of 30,000 h^−1^. The age and fully dried samples were labeled as 1.2-a. The NH_3_-SCR catalytic activity of the aged samples was investigated in a micro fixed-bed reactor.

## Results and discussion

### Catalytic performance

**FIGURE 2:**
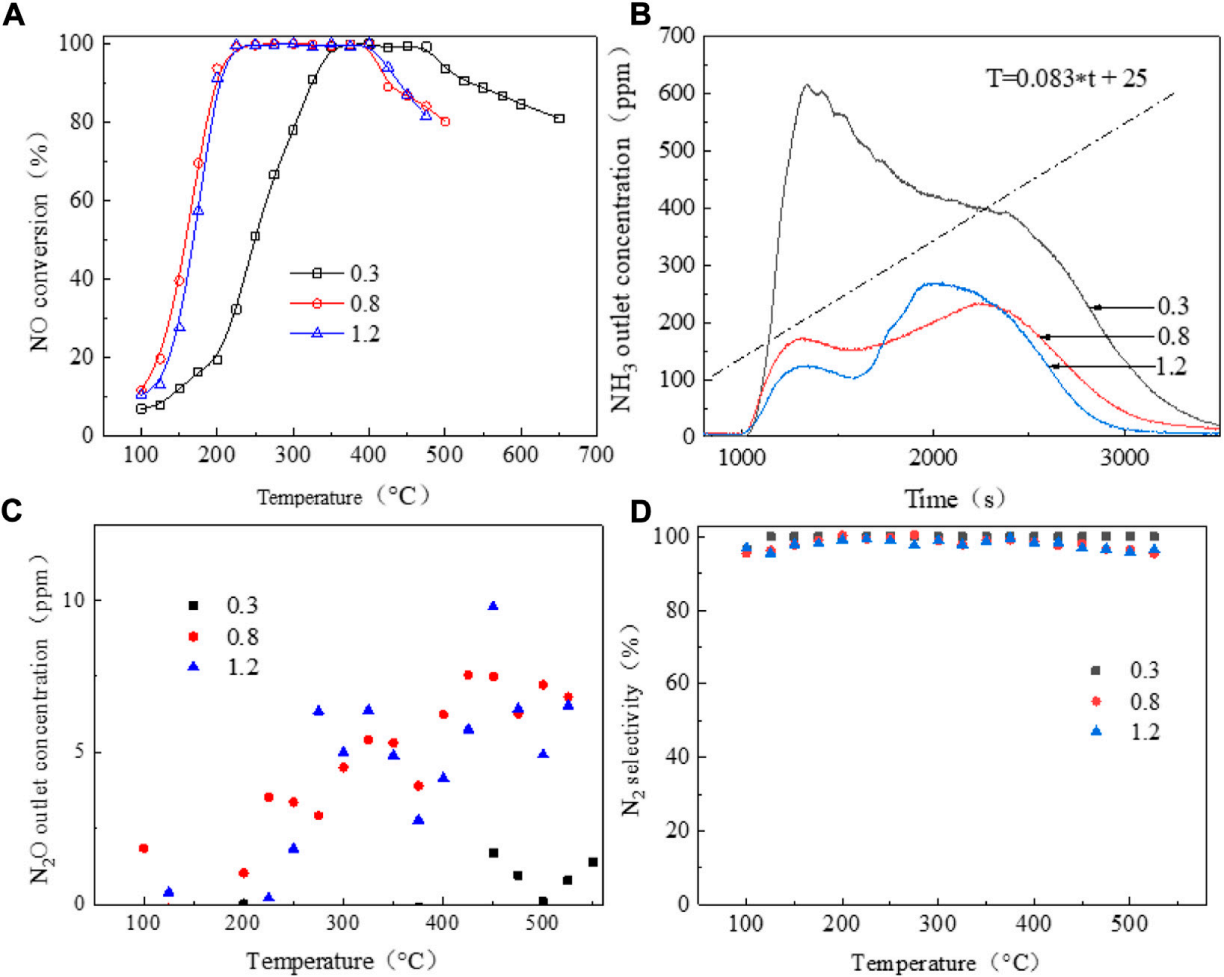
NH_3_-SCR performance of a series of copper-based SCR catalysts. **(A)** No conversion; **(B)** NH_3_ outlet concentration; **(C)** N_2_O outlet concentration; **(D)** N_2_ selectivity.

Figure 2A shows the NO conversion curves of the series copper-based SAPO-18/SAPO-34 catalyst in the standard NH_3_-SCR reaction. With increasing copper content, the ion exchange sites on which copper ions bonded to the molecular sieve as well as the formed active species are generally believed to change, with the temperature window shifting from higher to lower temperatures. The common copper active species in Cu-based zeolite SCR catalysts include Cu^2+^, [Cu(OH)]^+^, Cu-O-Cu, CuOx, etc. (Borfecchia et al., 2015; Shan et al., 2019; Liu et al., 2020; Khurana et al., 2022). Figure 2A shows that when the copper content increased from 0.3wt% to 0.8wt%, the range of the active temperature window T_90_ (the temperature at which the NO conversion rate is 90%) widened from 325–525°C to 170–425°C. Accordingly, the NO ignition temperature T_50_ (the temperature at which the NO conversion rate is 50%) decreased from 250°C to 160°C. Kwak et al. (2012) reported that at low copper content, the active component copper preferentially occupied the sites in the D6R cage, while some copper ions migrated to the CHA cage when the copper content increased. At a copper content of 1.2wt%, the low-temperature performance of the catalyst decreased slightly and the T_50_ temperature increased to about 165°C compared to 0.8wt%, and the high-temperature activity increased slightly, with the temperature window widening to 175–435°C. All Cu-based SAPO-18/SAPO-34 catalysts showed excellent nitrogen selectivity of close to 100%, as shown in Figure 2D.

Figure 2B shows the outlet ammonia concentrations of the series of copper-based SAPO-18/SAPO-34 catalysts during the activity test. In general, the SAPO-18/SAPO-34 intergrown zeolite showed poor ammonia storage performance, with ammonia escape concentrated at 100°C–300°C. At temperatures above 100°C, the adsorbed NH_3_ showed excessive desorption. Due to the lower temperature and the poor catalyst activity, NH_3_ could not fully participate in the selective catalytic reduction reaction, resulting in a sharp increase in its desorption capacity. When the number of catalytic active centers was small, this situation was particularly prominent. The amount of NH_3_ escape ranked from high to low was as follows: under 0.3wt%> 0.8wt%> 1.2wt%. N_2_O production showed the opposite trends as those for ammonia escape, as shown in Figure 2C. At lower copper content, less secondary pollutant N_2_O was generated (Isapour et al., 2022), which may be related to the species of CuOX crystal cluster; however, the overall production was <10 PPM, which met the relevant limit requirements for national emission standards.

### Physicochemical properties of the intergrown zeolite

**FIGURE 3:**
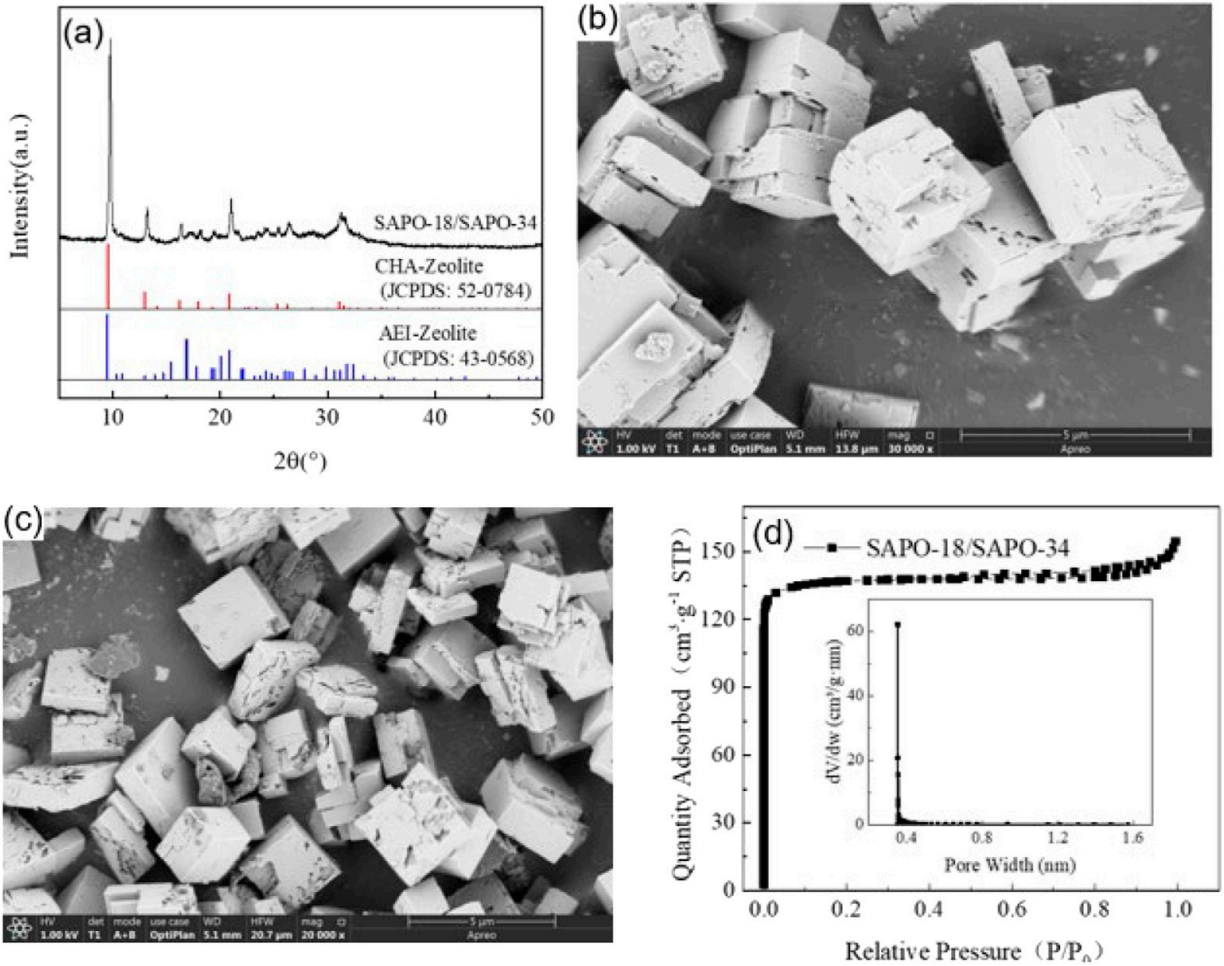
**(A)** XRD patterns; SEM photo of a SAPO-34/SAPO-18 intergrown zeolite: **(B)** fresh sample (30,000x) and **(C)** aged sample (20,000x). **(D)** N_2_ adsorption-desorption isotherms and porosity distribution curve of a SAPO-34/SAPO-18 intergrown zeolite.

Figure 3A shows the XRD pattern of the SAPO-34/SAPO-18 intergrown zeolite. The characteristic diffraction peaks are attributed to 2θ = 9.79°, 13.22°, 16.41°, 18.18°, 19.51°, 21.05°, 23.56°, 24.47°, 25.47°, 26.45°, 31.27°, and 31.61°, respectively. By comparison to the standard spectrum diagram in Figure 3A, the characteristic diffraction peaks of the SAPO-34 zeolite were attributed to 2θ = 9.64°, 13.08°, 14.20°, 16.28°, 18.05°, 19.35°, 20.94°, while the characteristic diffraction peaks of SAPO-18 zeolite were attributed to 2θ = 9.60°, 13.08°, 14.78°, 15.48°, 16.97°, 19.28°, 19.49°, 20.12°, 24.85°, and 26.11°. In contrast, the characteristic diffraction peaks of the SAPO-34/SAPO-18 intergrown zeolite were wide and weak in the range of 2θ = 16–18° and 30–33°, which were not observed in the above two single crystal zeolites. The ellipsoidal CHA structure and the pear-shaped AEI structure have similar skeleton topologies and the hexagonal prism cage (D6R) is key to the connection of the two lattices (Zhao et al., 2017). Where the D6R cage of CHA structural zeolite is straight and parallel, AEI structural zeolite shows a cross-oblique and parallel distribution. Therefore, the SAPO-34/SAPO-18 intergrown zeolite was more inclined to cross-stack on the cubic bulk crystal, with the microstructure shown in Figures 3B,C.

Figure 3B shows the microstructure photos of the SAPO-34/SAPO-18 intergrown zeolite, displaying generally irregular cross-stacked cube blocks. The crystal size is 3–5 μm, and the morphology obviously differs from those of SAPO-34 and SAPO-18 zeolites, which intuitively confirmed the formation of a eutectic structure. The intergrown crystal surface of SAPO-34/SAPO-18 was not smooth, but rather showed defects and damage, possibly because the use of the concentrated sol-gel system to prepare the intergrown zeolite affected the nucleation and growth process. The industrial pure-grade silicon and aluminum sources may have also contributed to the irregular morphology. After low-temperature hydrothermal aging, the defects on the SAPO-34/SAPO-18 crystal surface expanded and showed a tendency for fragmentation, indicating that its structure was damaged, as shown in Figure 3C.

Figure 3D presents the nitrogen physical adsorption test results of the SAPO-34/SAPO-18 intergrown zeolite. The nitrogen isotherm absorption/desorption curve of the intergrown zeolite showed type I isotherm characteristics, as defined by IUPAC, and had a type H4 hysteresis loop. That is, in the interval 0 < P/P0 < 0.01, the adsorption curve rose sharply with increasing relative pressure, indicating that the intergrown zeolite had structural characteristics of microporous materials (Lu et al., 2022). According to the gap width distribution curve, the median pore size was approximately 0.36 nm (Horvath-Kawazoe method); the narrow and sharp distribution reflected the regular lattice and orderly pore distribution of the intergrown zeolite. Moreover, the two-phase symbiosis did not significantly change the skeleton types of the CHA and AEI structural zeolites.

The XRF test results in Table 1 show an elemental composition of SAPO-34/SAPO-18 intergrown zeolite of SiO_2_:P_2_O_5_: Al_2_O_3_ = 1:3.74:3.81, which is related to the use of phosphoric acid-organic amine as template agent. The intergrown zeolite had low copper ion exchange efficiency and the copper content was only 0.3wt% after constant exchange at 80°C for 8 h. The copper content increased to 0.8wt% after two more exchanges. After three exchanges, the copper content loading rate was only 1.2 wt%. However, with increasing rounds of ion exchange, the specific surface area of the corresponding zeolite catalyst decreased significantly. The specific surface area of the SAPO-34/SAPO-18 intergrown zeolite was as high as 557 m^2^ g^−1^ and decreased to 487 m^2^ g^−1^ after three rounds of copper ion exchange. This may occur due to damage to the SAPO-34/SAPO-18 intergrown zeolite structure in the hot water environment used in ion exchange.

**TABLE 1:** XRF and BET results of SAPO-34/SAPO-18 zeolite and its catalysts.

Sample number	Fraction mole ratio	Copper content (wt%)	Specific surface area (m^2^·g^−1^)	Median pore diameter (Ǻ)
SAPO-18/SAPO-34	SiO_2_:P_2_O_5_: Al_2_O_3_ = 1:3.74:3.81	0	557	3.60
0.3	—	0.3	523	3.60
0.8	—	0.8	508	3.61
1.2	—	1.2	487	3.61

**FIGURE 4:**
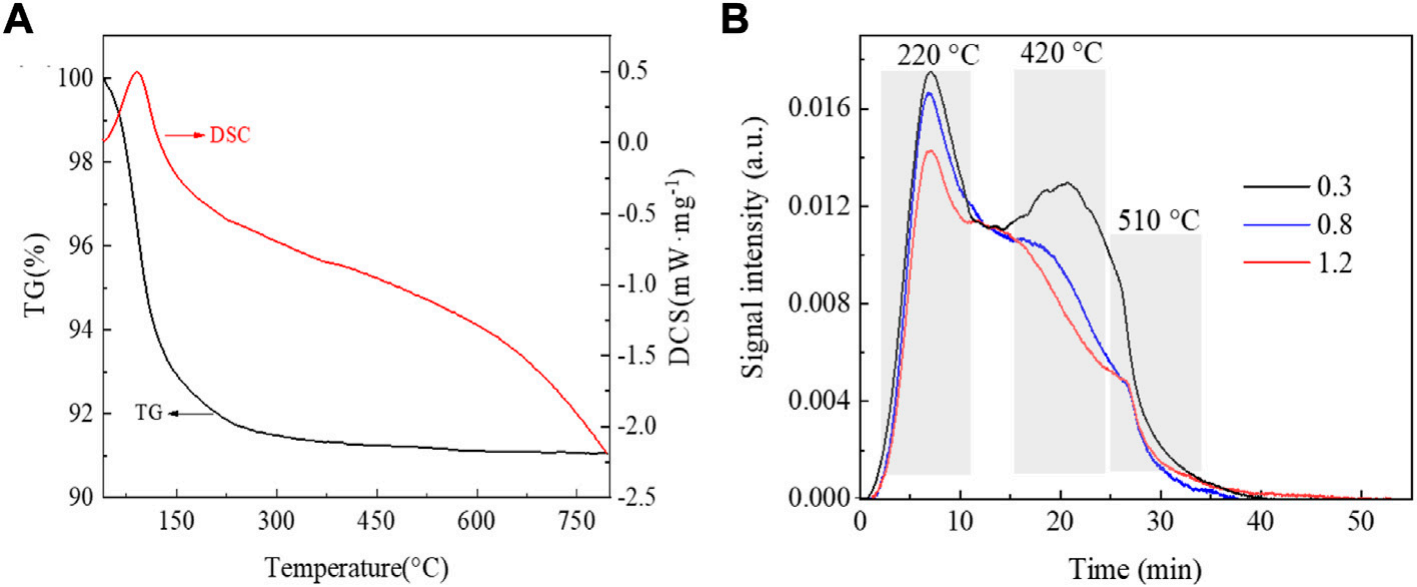
**(A)** TG-DSC curve of a SAPO-34/SAPO-18 intergrown zeolite. **(B)** NH_3_-TPD curves of the series of copper-based SCR catalysts.

Figure 4A shows the test results of the synchronous thermal analysis of the SAPO-34/SAPO-18 intergrown zeolite. The weight loss curve (TG) shows two main weight loss intervals at 40–150°C and 150–300°C and a weight loss rate of about 9 wt%. Below 150°C, the weight loss is obvious (up to 7 wt%), which is related to the rapid evaporation of excessive free water adsorbed by the zeolite. The heat curve (DSC) also reflects the water evaporation and heat absorption at this stage. With increasing temperature, the remaining water bound in the zeolite begins to volatilize and the organic amine template agent undergoes thermal decomposition with heating. The significant slowing of mass and heat changes at 150–300°C were attributed to the new phosphoric acid-organic amine template used in the SAPO-34/SAPO-18 synthesis process effectively avoiding the excessive use of template agent compared to the traditional method. The use of phosphoric acid-organic amine as a structure-diverting agent allowed the accurate and efficient use of organic amine molecules, thus improving environmental protection and economic benefits.

### Ammonia adsorption characteristics

The surface acidity of zeolite catalysts is generally believed to have an important influence on NH_3_-SCR performance. The surface acidity and amount of acid in zeolite SCR catalysts are usually characterized by NH_3_-TPD. Figure 4B shows the temperature-programmed ammonia desorption curves of the series of copper-based SAPO-34/SAPO-18 zeolite catalysts. The three main characteristic peaks were attributed to weak, medium-strong, and strong acid sites, respectively (Pérez-Uriarte et al., 2016; Xu et al., 2017; Shin et al., 2018). The weak acid site at low temperatures corresponded to a weak Lewis acid (T = 220°C), the medium strong acid site at middle temperatures corresponds to strong Lewis acids and some active copper species sites (T = 420°C), and the strong acid adsorption site at high temperatures corresponded to a Brønsted acid (T = 510°C). As shown in Figure 4B, increasing copper content was associated with a decreased acid content of the zeolite catalyst and acid peak intensity to varying degrees. The Brønsted acid position was particularly obvious. Regarding the main factors affecting Brønsted acid sites, 1) during the ion exchange, Cu occupies the hydroxyl site of Si-OH-Al in the six-member ring of zeolite to form Cu^2+^, with Brønsted acid sites decreasing accordingly (Villamaina et al., 2019). 2) During ion exchange reactions, the high-temperature water environment damages the structure of the SAPO-34/SAPO-18 zeolite, leading to a significant reduction of Brønsted acid sites (Liu et al., 2022). 3) The copper loading amount in the zeolite is exceeded and the active components mainly exist as [Cu(OH)]^+^ and CuOx clusters (Lee et al., 2021). The generation of CuO may lead to the dealuminization of the zeolite skeleton; that is, the destruction of zeolite Brønsted acid (Si-OH-Al) (Di Iorio et al., 2015; Millan et al., 2021; Negri et al., 2021). Based on the above factors, we further investigated the effect of low-temperature hydrothermal aging treatment on the performance of the NH_3_-SCR catalyst.

### Low-temperature hydrothermal stability

**FIGURE 5:**
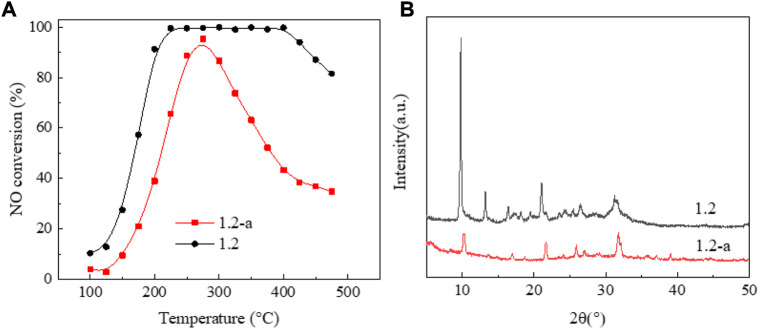
Performance changes of the 1.2 wt% copper content catalyst before and after low-temperature hydrothermal aging. **(A)** NO conversion. **(B)** XRD patterns.

**FIGURE 6:**
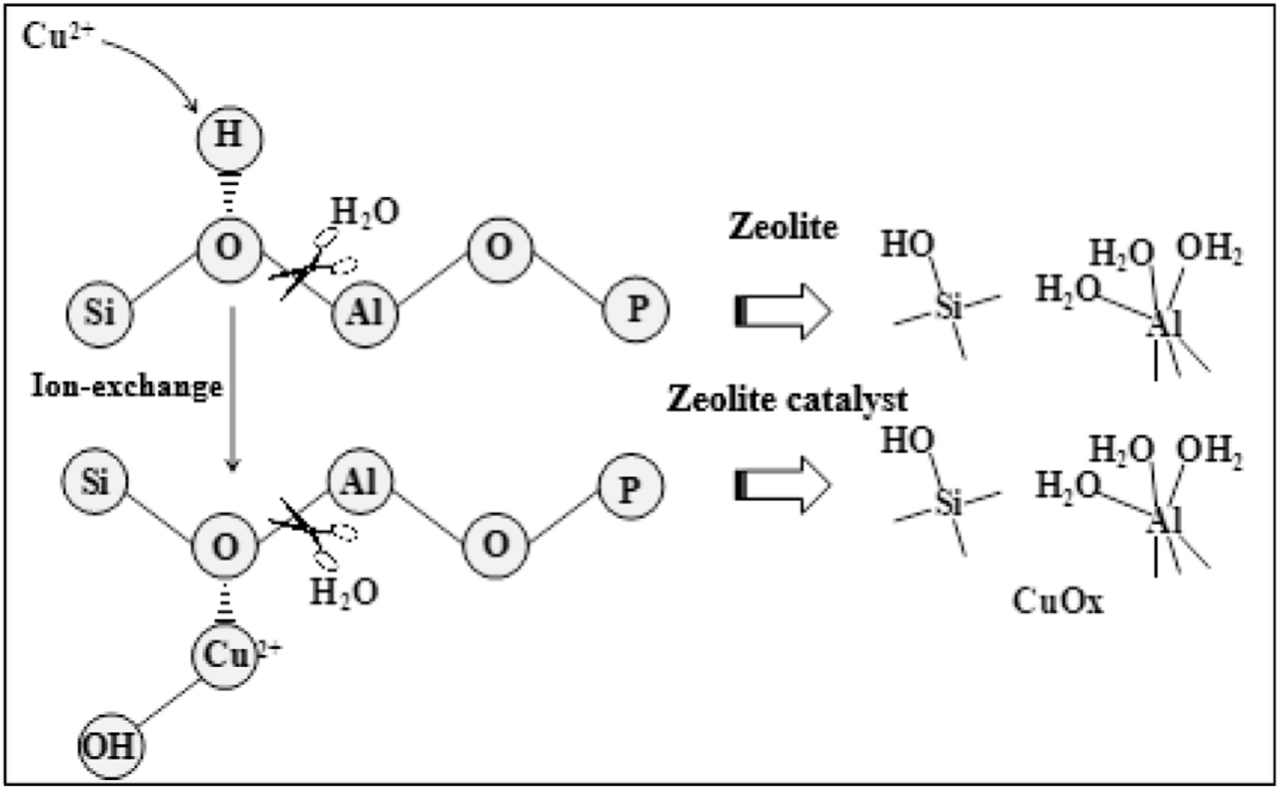
Schematic diagram of the destruction of zeolite and its catalyst by low-temperature hydrothermal aging treatment.

Figure 5A shows the NO conversion curve of a 1.2 wt% sample before and after low-temperature hydrothermal aging. Figure 5A shows significantly decreased catalyst activity with aging. Moreover, the temperature window basically disappears, and the NO ignition temperature exceeds 200°C. The results of crystal phase structure characterization Figure 5B demonstrated that, after aging, the catalyst presents an amorphous state and the characteristic diffraction peak of SAPO-34/SAPO-18 zeolite almost completely disappears. The above results suggest that the crystal phase structure of the copper-based SAPO-34/SAPO-18 zeolite catalyst was destroyed after low-temperature hydrothermal aging treatment, leading to irreversible inactivation (Woo et al., 2018) (Figure 6).

Ma et al. (2020) reported that the Si-O(H)-Al bond of SAPO-34 zeolite was prone to hydrolysis in low-temperature hydrothermal environments. Woo et al. (2020) believed that the hydrolysis first formed Si (2Al) (2OH) and Si (3Al) (OH), which were finally transformed into silicon clusters. Gao et al. (2013) reported that SAPO-34 zeolite structures with more Si-O(H)-Al bonds showed more serious hydrolysis damage. Wang et al. (2019) studied the effect of SAPO-34 zeolite hydrolysis on active species, in which Cu(OH)^+^ was transformed into spinel-structured CuAl_2_O_4_, with significantly decreased catalytic activity. Leistner et al. (2015) confirmed the loss of the active Cu^2+^ species lost after the water vapor treatment of Cu-SAPO-34. Zhang et al. (2022b) proposed that the hydrolysis of the Si-O(H)-Al bond and the loss of active copper species jointly induced Cu/SAPO-34 inactivation, with inactivation the easier under a larger proportion of the two.

## Conclusion

First, SAPO-18/SAPO-34 intergrown zeolite was prepared using phosphoric acid-organic amine, which has a unique crystal phase structure and microstructure, displaying the typical adsorption characteristics of zeolite microporous materials. Phosphoric acid-organic amines act as both a phosphorus source and a structure-diverting agent. The excessive use of structure-diverting agents can be avoided by use of a synthetic stoichiometric ratio. TG-DSC test results did not show an obvious thermal decomposition phenomenon of organic amines. Secondly, with increasing active component content, the state of copper species changed due to migration, and the active temperature window of the Cu-based SAPO-18/SAPO-34 catalyst shifted toward low temperatures. At copper contents <0.8wt%, the T_50_ was 160°C, and the T_90_ range was 170–425°C, showing the optimal performance. Finally, the SAPO-18/SAPO-34 intergrown zeolite showed three main ammonia adsorption sites, and the Brønsted acid sites of the zeolite carrier were lost due to copper occupying the exchange sites during the ion exchange. However, a more important incentive is the structural damage of SAPO zeolite in the low-temperature hydrothermal process. After low-temperature hydrothermal aging treatment, the temperature window of the 1.2wt% Cu content sample almost disappeared, with the crystal phase structure seriously damaged.

## Data Availability

The raw data supporting the conclusion of this article will be made available by the authors without undue reservation.
